# Supplementary material for: Synthesis of Graphene Oxide from Sugarcane Dry Leaves by Two-Stage Pyrolysis
Source: Molecules. 2023 Apr 10;28(8):3329. doi: 10.3390/molecules28083329 (PMC10140955; doi:10.3390/molecules28083329)
Supplement: Supplementary file 1 [file molecules-28-03329-s001.zip › molecules-2308281-supplementary.pdf]

Supplementary information

Table S1. Comparative analysis of GO Synthesis methods and properties reported in the literature.

Synthesis method	Carbon precursor	Reaction time	Reaction temperature (°C)	Oxidizing / Reducing agents	Functional groups	I_D/I_G ratio	Interlayer spacing '*d*' (nm)	Reference
Improved Hummers'	Graphite flake	12 h	> 50	H_2SO_4 / H_3PO_4, $KMnO_4$	O-H, C=O, C=C, C-O	NA	0.95	[11]
Modified Hummers'	Graphite flake	13 h	> 35	$NaNO_3$, H_2SO_4, $KMnO_4$, $K_3Fe(CN)_6$	O-H, CH_2, C=C, C=O, C-O, C-OH	NA	0.77	[80]
Hummers' and improved Hummers' with purification	Graphite powder	7.5 days	40	$NaNO_3$, H_2SO_4, $KMnO_4$	C-O-C, C-O, C=C, C=O, O-H	NA	0.81	[12]
Improved Hummers' with purification	Graphite powder	7.5 days	40	H_2SO_4, $KMnO_4$, H_2O_2	C-O-C, C-O, C=C, C=O, O-H	0.61	0.831	[81]
Modified Hummers'	Graphite flake	5 h	> 35	H_2SO_4, $KMnO_4$, K_2FeO_4	-OH, CH_2, C=C, C=O, C-O, C-O-C	GO1 -1.07 GO2-0.94	GO1-0.83 GO2-0.81	[82]
Pyrolysis and Hummers' method	Rice straw	4.5 h	450 (Pyrolysis), 45 (Hummer's method)	H_2SO_4, $KMnO_4$,	C=C, C=O, O-H, C=C, C-N, C-H, C-N	NA	NA	[7]
Two-stage pyrolysis	Sugarcane dry leaves	9 h	800	H_2SO_4	-OH, CH_2, C-O, C=C, C=H, C=O	0.92	0.71	This study

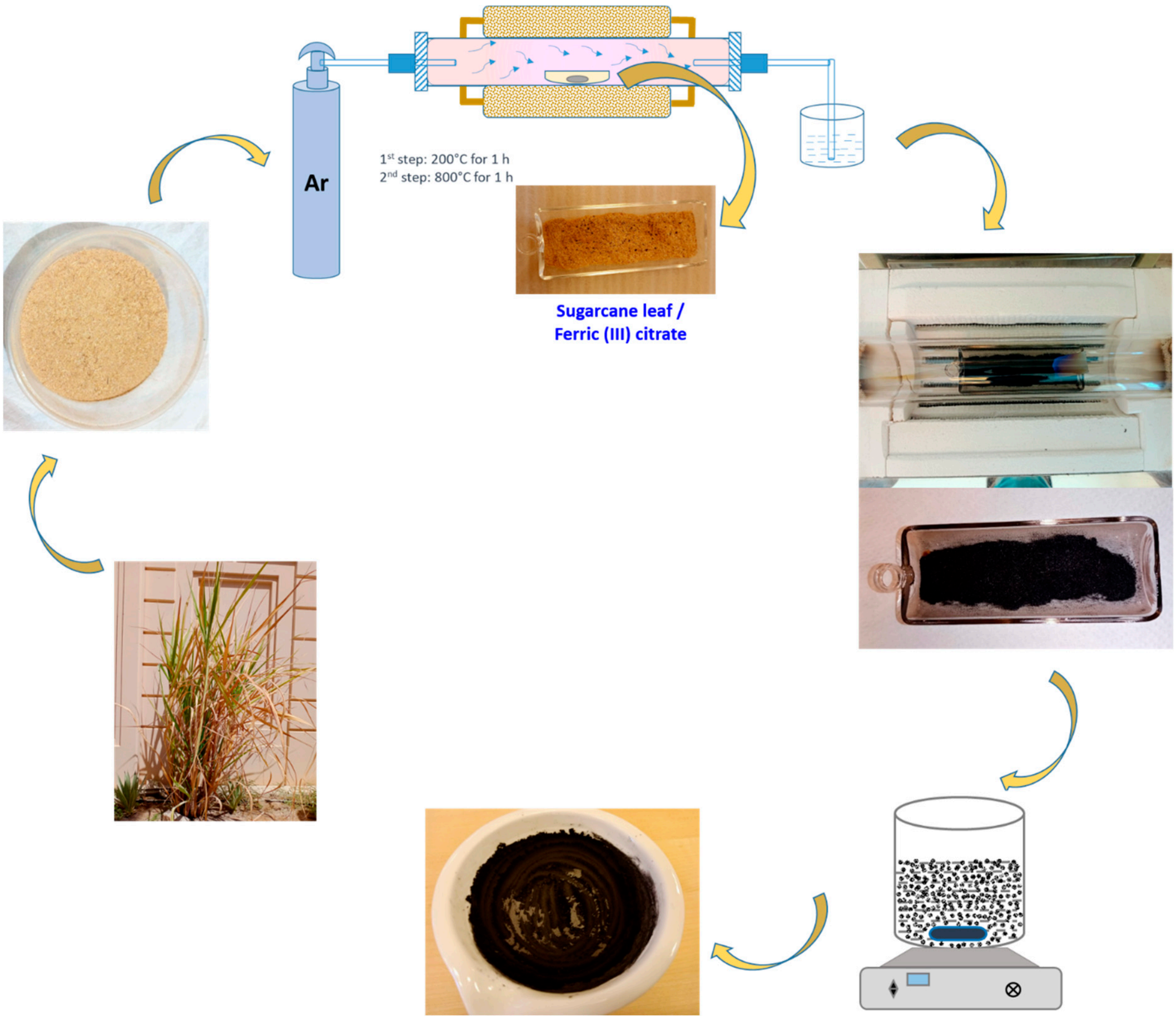

Scheme S1. The processing steps for the preparation of GO from sugarcane dry leaves.
